# Estradiol cypionate modulates immunological response during sepsis

**DOI:** 10.1186/cc12982

**Published:** 2013-11-05

**Authors:** Luiz E da Silva, Angelita M Stabile, Marcel E Batalhão, Evelin C Cárnio

**Affiliations:** 1 College of Nursing at Ribeirão Preto, São Paulo, Brazil

## Background

Sepsis and its common complication septic shock are generally induced by the action of lipopolysaccharide (LPS) and characterized by peripheral arteriolar vasodilatation that results in hypotension and inadequate tissue perfusion. During sepsis, secretion occurs of large amounts of inflammatory mediators such as nitric oxide (NO), interleukin 1 (IL-1) and TNFα that will modulate the inflammatory response. One significant finding in clinics is that men and women respond differently to sepsis, with better prognosis related to women [1].

## Materials and methods

Male and female (ovariectomized and sham surgery) rats were injected intraperitoneally (i.p.) for three consecutive days with ECP 40 µg/kg or vehicle. On the third day, after ECP injection, rats receive i.p. injection of 10 mg/kg bacterial LPS or saline solution. Plasma was collected 2, 4 and 6 hours after LPS for NO and cytokine measurements.

## Results

Administration of LPS increased the NO plasma concentration in males and females (2, 4 and 6 hours). ECP pretreatment decreased the NO concentration in sham females at 4 and 6 hours; conversely, it increased nitrate levels in ovariectomized and in males at 4 and 6 hours. IL-1 plasma concentration was increased in the three groups after LPS administration at 2 and 4 hours and in Sham at 6 hours; ECP pretreatment decreased IL-1 plasma concentration in all groups at 2 hours. LPS administration also increased TNFα plasma concentration at 2, 4 and 6 hours in the three groups; ECP pretreatment inhibited the increase of TNFα at 2 hours in three groups.

## Conclusions

Our results indicate that estradiol may have proinflammatory or anti-inflammatory actions depending on the gender and the mediator evaluated; this balance in mediator secretion may be protective and explain in part the better outcomes of woman during sepsis.
